# Polysaccharides from *Ganoderma lucidum* attenuate microglia-mediated neuroinflammation and modulate microglial phagocytosis and behavioural response

**DOI:** 10.1186/s12974-017-0839-0

**Published:** 2017-03-24

**Authors:** Qing Cai, Yuanyuan Li, Gang Pei

**Affiliations:** 1 State Key Laboratory of Cell Biology, Institute of Biochemistry and Cell Biology, Shanghai Institutes for Biological Sciences, Chinese Academy of Sciences, 320 Yueyang Road, Shanghai, 200031 China; 2 Graduate School, University of Chinese Academy of Sciences, Chinese Academy of Sciences, 320 Yueyang Road, Shanghai, 200031 China; 3 School of Life Science and Technology, and the Collaborative Innovation Center for Brain Science, Tongji University, Shanghai, 200092 China

**Keywords:** Microglia, *Ganoderma lucidum* polysaccharides, Neuroinflammation, Behavioural response, Amyloid beta

## Abstract

**Background:**

*Ganoderma lucidum* (GL) has been widely used in Asian countries for hundreds of years to promote health and longevity. The pharmacological functions of which had been classified, including the activation of innate immune responses, suppression of tumour and modulation of cell proliferations. Effective fractions of *Ganoderma lucidum* polysaccharides (GLP) had already been reported to regulate the immune system. Nevertheless, the role of GLP in the microglia-mediated neuroinflammation has not been sufficiently elucidated. Further, GLP effect on microglial behavioural modulations in correlation with the inflammatory responses remains to be unravelled. The aim of this work was to quantitatively analyse the contributions of GLP on microglia.

**Methods:**

The BV2 microglia and primary mouse microglia were stimulated by lipopolysaccharides (LPS) and amyloid beta_42_ (Aβ_42_) oligomer, respectively. Investigation on the effect of GLP was carried by quantitative determination of the microglial pro- and anti-inflammatory cytokine expressions and behavioural modulations including migration, morphology and phagocytosis. Analysis of microglial morphology and phagocytosis modulations was confirmed in the zebrafish brain.

**Results:**

Quantitative results revealed that GLP down-regulates LPS- or Aβ-induced pro-inflammatory cytokines and promotes anti-inflammatory cytokine expressions in BV-2 and primary microglia. In addition, GLP attenuates inflammation-related microglial migration, morphological alterations and phagocytosis probabilities. We also showed that modulations of microglial behavioural responses were associated with MCP-1 and C1q expressions.

**Conclusions:**

Overall, our study provides an insight into the GLP regulation of LPS- and Aβ-induced neuroinflammation and serves an implication that the neuroprotective function of GLP might be achieved through modulation of microglial inflammatory and behavioural responses.

**Electronic supplementary material:**

The online version of this article (doi:10.1186/s12974-017-0839-0) contains supplementary material, which is available to authorized users.

## Background

*Ganoderma lucidum* is a well-known herb used in the traditional Chinese medicine to promote longevity and is beneficial for general health [1, 2]. In recent years, the extract of *Ganoderma lucidum* (GL) has been isolated [3–5] and frequently used in medications as well as in dietary supplements. The constituents of GL include mainly ergosterol, triterpenoids, unsaturated fatty acids and polysaccharides. Amongst all, polysaccharides are the major pharmacologically active ingredient. The effects of GL extracts had been related to the promoted innate immune responses, suppression of cancer cell migration, as well as modulations of cell proliferations [6–8]. In recent years, studies have shown that GL exhibited neuroprotective effect and significantly attenuated amyloid beta (Aβ) peptide-induced neurotoxicity [9]. In addition, evidence showed that pre-administration of GL spores to rat might also protect the hippocampus from oxidative damages [10]. All of these provided positive implications for GL in the treatment of Alzheimer’s disease (AD). Nevertheless, there have not been sufficient studies in the biochemical mechanism to which GLP might target AD.

The aetiology of AD is of complex mechanisms and not yet fully resolved. Two hallmarks characterising this neurodegenerative disease are the aggregation of Aβ leading to senile plaques and the progressive cognitive impairments [11]. In the central nervous system (CNS), deposition of Aβ results into the activation of microglia, the resident immune cells and thus neuroinflammation [12]. Activated microglia release pro-inflammatory cytokines and neurotoxic mediators with altered cell behaviours, which may be characterised by the microglial morphology, migration and phagocytosis [13]. A positive feedback from microglial phagocytosis is the removal of dead neurones and neuronal debris, which in turn contributes to the attenuation of inflammatory stress. However, prolonged activation by Toll-like receptor (TLR) agonists, such as lipopolysaccharides (LPS), Aβ and lipoteichoic acid, may result into aberrant phagocytosis process [14, 15]. Under such conditions, microglia target on live neurones, neuronal progenitor cells (NPC) and glioma cells, all of which leads to neuronal loss in the CNS [14].

In the present study, we aimed to investigate the effect of GLP on the LPS- and Aβ-induced microglial behavioural changes. Apart from the pro-inflammatory mediators, chemokines such as MCP-1 also accumulate as a result of neuroinflammation. MCP-1 over-expression has been detected in many neurodegenerative diseases [16–18]. In the AD brain, the function of MCP-1 is related to cell movement and initiates monocyte accumulation at the site of Aβ deposition [19–21]. The up-regulation of MCP-1 expression may contribute to the chronic inflammation [22]. Our results revealed that GLP reduced the pro-inflammatory cytokines and MCP-1 expressions with a tendency to promote anti-inflammatory cytokine levels. We also demonstrated that GLP modulation of microglial behavioural changes in vitro was associated to MCP-1 expressions. Finally, we confirmed the GLP-modulated microglial behavioural changes in vivo.

## Methods

### Animals

The current study was conducted in strict accordance with the guidelines of the Institute of Biochemistry and Cell Biology, Chinese Academy of Sciences. All experimental protocols in the study were approved and overseen by the Animal Care and Use Committee of the Shanghai Institute of Biochemistry and Cell Biology, Chinese Academy of Sciences. All mice (C57BL/6) were maintained in the pathogen-free conditions.

### *Ganoderma lucidum* polysaccharides

The GL polysaccharides were provided by Shanghai Lugu Pharmaceuticals and were extracted from the dried conidial powder of GL according to previous protocol [23]. In brief, 1.35 kg of *G. lucidum*-dried conidial powder was defatted with 95% EtOH for 1 week, followed by 5-h boiling water extraction procedure for six repeated times. The supernatant was combined, concentrated and centrifuged. To the concentrated supernatant, three volumes of 95% EtOH were added to precipitate the crude polysaccharides, CPW (28.2 g, 2%). CPW (7 g) was then fractionated and eluted using distilled water to obtain the water extracts (3.25 g). This water extracts were further purified on a Sephacryl S-300 column (2.6 cm × 100 cm) and eluted with 0.2 M NaCl to obtain GLP. By high-performance gel permeation chromatography (HPGPC) method, the relative molecular weight of GLP was estimated at approximately 15 kDa. After careful examinations by the Lowry method and m-hydroxydiphenyl method, it was confirmed the polysaccharides contained no trace of protein and uronic acid. One single preparation of GLP was performed for all experiments.

### Aβ_42_ oligomer preparation

The Aβ_42_ oligomers were prepared based on protocols by Stine [24]. In brief, the HFIP (hexafluoroisopropanol)-treated Aβ_42_ peptides (Anaspec) were resuspended in dimethyl sulfoxide followed by dilution to 100 mM in Ham’s F12. After incubation for 24 h at 4 °C, the soluble Aβ_42_ oligomers were obtained and centrifuged for 10 min at 14,000*g*. The integrity of Aβ_42_ oligomers were previously validated by atomic force electromicroscopy and western blot [25]. In addition, dot blots were performed to confirmation the oligomeric and fibrillar forms of Aβ_42_ (Additional file 1: Figure S2).

### Cell culture and treatment

BV2 cell lines were cultured and maintained in Dubelcco’s Minimal Essential Medium (DMEM), with 10% fetal bovine serum (FBS) supplement and 100 U/ml penicillin and 0.1 mg/ml streptomycin. Primary microglia were prepared from wild-type C57BL/6 mice on postnatal day 1. In brief, the mice’ cortices and hippocampi were dissected and the meninges were carefully removed. The combined cortical and hippocampal tissues were dissociated into single cells by gentle scissoring and pipetting. The resultant suspension of cells was seeded to a T75 flask, cultured in DMEM supplemented with 10% FBS and 100 U/ml penicillin and 0.1 mg/ml streptomycin. After 7–10 days, microglial cells were isolated from the astrocyte monolayer sheet by shaking.

In all in vitro assays, BV2 cells and primary microglia were pre-treated with GLP at various concentrations for 2 h (1 ng/ml–1 μg/ml for BV2, 0.3 ng/ml–0.1 μg/ml for primary microglia). At the end of pre-treatment, 1 μg/ml LPS (055:B5, Sigma) or 10 μM Aβ_42_ oligomer was added for further 24 h.

### Quantitative real-time reverse transcription polymerase chain reaction (qRT-PCR)

To analysis the mRNA expression, BV2 cells were seeded into 96-well plates and primary microglia into 12-well plates at appropriate densities. After the cell treatment, total RNA was extracted using the TRI Reagent® (Sigma) according to the manufacturer’s instructions. RNA purity and integrity were assessed with NanoDrop 1000 Spectrophotometer (Thermo Scientific). A two-step first-strand cDNA synthesis reaction was performed using TIANScript M-MLV kit (TIANGEN) following the manufacturer’s protocols. Addition of rRNasin® (Recombinant rRNasin® Ribonuclease Inhibitor, Promega) was used in the synthesis. The expression of mRNAs was determined by quantitative real-time PCR using the 2× HotStart SYBR Green qRT-PCR Master Mix kit (ExCell). In brief, the reaction consisted of 4 μl of pre-diluted cDNA in a total volume of 25 μl supermix containing 0.25 μM primers. The reaction parameters were as follows: 95 °C for 10 min; 95 °C for 30 s, 40 cycle; 60 °C for 30 s; 72 °C for 30 s. An additional cycle was performed for evaluation of primer’s dissociation curve: 95 °C for 1 min, 60 °C for 30 s and 95 °C for 30 s. Each cDNA sample was amplified in duplicates. Primer sequences used in the experiments are listed in Table 1.

**Table 1:** Primers used for qPCR

Gene	Forward	Reverse
IL-1β	GTTGACGGACCCCAAAAGAT	AAGGTCCACGGGAAAGACAC
IL-6	TAGTCCTTCCTACCCCAATTTCC	TTGGTCCTTAGCCACTCCTTC
iNOS	CCCTTCAATGGTTGGTACATGG	ACATTGATCTCCGTGACAGCC
Arg1	GAACACGGCAGTGGCTTTAAC	TGCTTAGCTCTGTCTGCTTTGC
TGFβ	CACTGATACGCCTGAGTG	GTGAGCGCTGAATCGAAA
MCP-1	ATGCAGGTCCCTGTCATGCTTC	TTCTGATCTCATTTGGTTCCGA
C1q	GGCTGGAGCATCCAGTTTGA	GTCATGGTCAGCACACAGGC

### ELISA determination of IL-6

The supernatants from each treatment were collected for ELISA assays. The supernatants were first centrifuged to remove cellular debris. Concentrations of IL-6 were determined using mouse-specific pre-coated ELISA kits (DAKEWE Biotech Company) according to the manufacturer’s instructions.

### Immunocytochemistry

Cultured mouse microglia were fixed in 4% paraformaldehyde (PFA) in PBS at room temperature for 15 min. Cells were then permeabilised and blocked non-specific bindings (1% BSA and 0.1% Triton X-100 in PBS) for 30 min. At room temperature, primary antibody anti-Iba1 (1:1000, WAKO) was applied for 2 h followed by secondary antibody Cy3-conjugated anti-rabbit IgG (1:1000) for 1 h. The immunocytochemistry staining images were captured using Olympus IX51 camera with an inverted laser (X-Cite® series120) and a 20×/0.45 Olympus objective.

### Scratch wound migration assay and morphological characterisation

BV2 cells were grown in 48-well plates till 70–80% confluent. The monolayer of cells was then wounded with a sterile 200-μl pipette tip in a straight line along the diameter of the well and washed three times with sterile PBS. The cells were grown for further 24 h allowing migration into the open scratched area. Images of cells were captured at 0 and 24 h after wounding, using a Zeiss A-Plan 5× objective (0.12 Ph0) and the Zeiss Observer Z1 microscope. Only single distinct cell was selected for morphological analysis. Dividing cells and those attached to each other were excluded. The absolute value of distance migrated by cells was quantified as the change in the perpendicular distance between the edge of the gap after 24 h. The value was then normalised to the 0 h starting measurement which represents “migration” $\left(\mathrm{Migration}=\frac{\mathrm{Distanc}\mathrm{e}{\;_{24\ \mathrm{hour}\mathrm{s}}}_{\hbox{--}}\mathrm{Distanc}{\mathrm{e}}_{0\ \mathrm{hour}}}{\mathrm{Distanc}{\mathrm{e}}_{0\ \mathrm{hour}}}\right)$. We used the Fiji ImageJ software (Version 2.0.0) to quantitatively characterise the morphology using the following parameters: cell area, perimeter, Feret’s diameter (the longest distance between any two points along the selection boundary) and circularity $\left(4\uppi \frac{\mathrm{Area}}{\mathrm{Perimete}{\mathrm{r}}^2}\right).$

### Phagocytosis assay

BV2 and primary microglia were plated into 24-well plates at appropriate densities to ensure the cells confluent to approximately 30,000 per well at the time of assay. Pre-treatment of GLP was carried out 2 h after plating and followed by stimulation with LPS or Aβ_42_. Phagocytosis assay were performed based on the protocol previously described [26]. Cell medium were changed to DMEM alone and allowed incubation for 30 min inside the 37 °C cell culture incubator. After this, the media were replaced by DMEM supplemented with 5% FBS under various treatment conditions for GLP and stimuli. The fluorescent latex beads (1 μM, L2778 Sigma) were pre-opsonised in 50% FBS and PBS. The pre-opsonised beads were loaded to the cells at concentrations of 50 beads per BV2 cell and 100 beads per primary microglia and incubated at 37 °C for 2 and 3 h, respectively. Negative controls were carried out with pre-treatment of Cytochalasin D for 30 min at 37 °C in order to prevent phagocytosis. At the end of incubation, the remaining beads were gently washed off the cells and fixed by 4% PFA at room temperature. Cells were then stained with DAPI (4',6-diamidino-2-phenylindole) at room temperature for 15 min, and images were captured under Zeiss Observer Z1 inverted microscope. For each well, DAPI, Cy3 and brightfield images were collected (*N* = 15–30) and analysis was done by Fiji ImageJ software (Version 2.0.0).

### Zebrafish and embryo maintenance

Zebrafish and embryo were raised in egg water in a 14-h light and 10-h dark cycle at 28.5 °C as described [27]. To inhibit pigmentation, 0.003% PTU was added to each samples at 24 h postfertilisation (hpf). The double transgenic zebrafish line *Tg*(*ApoE:GFP*, *Huc:mcherry*) was as previously described [28]. The zebrafish embryonic growth was first monitored in the presence of 1 μg/ml GLP from 12 hpf to 5 days postfertilisation (dpf). No developmental defects were observed (Fig. 4a–c). Subsequent experiments were performed pre-treating larva with GLP from 4 dpf for 24 h.

### In vivo time-lapse confocal imaging

1.5% low melting point agarose (Sigma) was prepared to fix the 5-dpf larvae alive without anaesthetics. A dorsal view of the optic tectum is placed. Living imagines were carried out in 1 μm/optical section (40–65 μm in depth) in a 5-min interval using Olympus FV1000 upright confocal microscope (473 nm, 543 nm; Japan) with a Zeiss 40×NA 0.80 water immersion.

### Image analysis

Resting and activated state morphology dynamic was analysed as previously described [28, 29]. The cell size, deformation speed and tip number of microglia were measured by the Fiji ImageJ (version 2.0.0).

### Cell viability assay

The proliferative potential of GLP was examined in the presence or absence of LPS. The BV2 cell proliferation was tested using the CellTiter-Glo luminescent cell viability assay (Promega) following the manufacturer’s instructions.

### Statistics

Statistical analysis was performed using GraphPad Prism 6 software (Graphpad Software, La Jolla, CA, USA). Results were analysed by unpaired two-tailed Student’s *t* test to determine the significance of the treatment sets. For comparisons between multiple groups, one-way ANOVA analysis with Tukey’s multiple comparison test was performed. All data were presented as mean ± SEM. *p* < 0.05 is considered to have significant difference.

## Results

### GLP attenuates LPS- or Aβ-induced inflammatory response in microglia

**Fig. 1:**
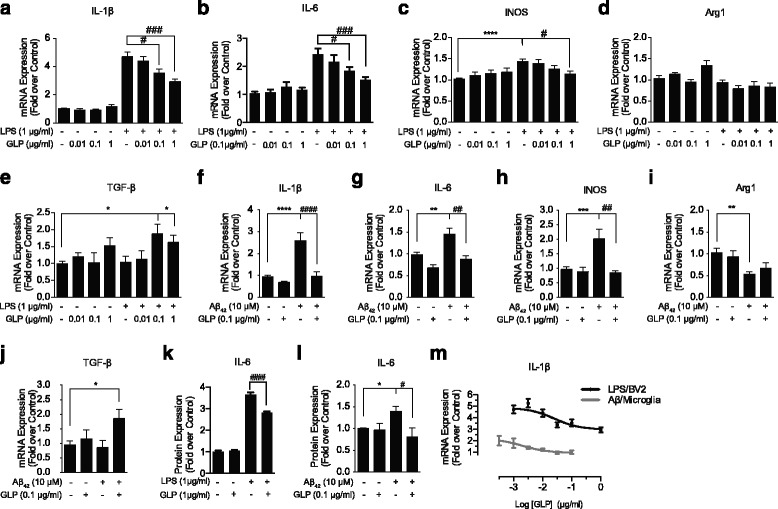
GLP attenuates LPS- and Aβ-induced inflammatory response in microglia. BV2 (**a**–**e**, **k**) and primary mouse microglia (**f**–**j**, **l**) were pre-treated with GLP for 2 h followed by LPS or Aβ stimulation for further 24 h. GLP inhibited the LPS- and Aβ-induced pro-inflammatory cytokine levels (IL-1β, IL-6 and iNOS) and promoted anti-inflammatory cytokine (TGFβ) mRNA expressions. The effect of GLP on IL-6 protein expression in BV2 (**k**) and primary microglia (**l**) was determined by ELISA. The IC_50_ values for GLP were calculated from concentration-response curves in BV2 and primary microglia (**m**). Values were expressed as mean ± SEM for at least three independent experiments, each performed in duplicates. One-way ANOVA with Tukey’s multiple comparison test revealed difference from untreated control (*) and LPS- or Aβ-stimulated condition (#) (compared with control: *p < 0.05, **p < 0.01, ***p < 0.005, and ****p < 0.0001; compared with LPS- or Aβ-stimulated condition: ^#^ *p* < 0.05, ^##^ *p* < 0.01, ^###^ *p* < 0.005, and ^####^ *p* < 0.0001)

In the previous report, GLP by an alternative extraction technique with different purity was shown to inhibit microglial activation [30]. In order to verify the integrity of the GLP used in the present study, we first examined the cytotoxicity and proliferative potential of GLP for 24 h (Additional file 1: Figure S1A). Further long-term (48, 72 and 96 h) incubations confirmed GLP did not affect cell growth (Additional file 1: Figure S1B). We then investigated the effect of GLP on the LPS-induced microglial activation (Fig. 1a–e). BV2 cells were pre-treated with GLP for 2 h followed by LPS stimulation. We showed that GLP inhibited LPS-induced pro-inflammatory cytokines IL-1β, IL-6 and inducible nitric oxide synthase (iNOS) expressions in a concentration-dependent manner (Fig. 1a–c). The LPS-induced IL-1β and IL-6 expressions were effectively reduced by GLP at 0.1 and 1 μg/ml, and iNOS expression down-regulated at 1 μg/ml. At lower concentrations (0.01 and 0.1 μg/ml), the effect of GLP on iNOS was not statistically significant. However, a concentration-dependent trend was observed. In addition, GLP up-regulated the anti-inflammatory cytokine TGFβ expression in the presence of LPS. It also revealed a tendency to increase Arg1 expression in the absence of LPS (Fig. 1d, e). In AD, the microglia-mediated neuroinflammation is stimulated by Aβ. Therefore, in this study, we used the soluble form of oligomeric Aβ_42_ and examined the role of GLP in primary mouse microglia (Fig. 1g–j, l). The results showed that GLP significantly inhibited the Aβ_42_-induced pro-inflammatory cytokines IL-1β, IL-6 and iNOS to approximate basal levels. In addition, GLP exhibited tendencies to promote anti-inflammatory cytokine expressions (Fig. 1i, j). Under Aβ_42_ activation, Arg1 expression was significantly reduced. It seemed that pre-treatment of GLP might potentially rescue its expression, but the effect was not significant. On the other hand, GLP promoted TGFβ expression in Aβ_42_-activated cells. We then confirmed these findings at protein levels by measuring the IL-6 protein expressions using ELISA (Fig. 1k, l). The IC_50_ values of GLP in both BV2 and primary microglia were analysed from concentration-response curves, the IL-1β expressions were determined as representatives. These values were calculated using GraphPad Prism software. The IC_50_ values were 25 ng/ml (LogIC_50_ = −1.598) and 2 ng/ml (LogIC_50_ = −2.704) for BV2 and primary microglia, respectively (Fig. 1m). Overall, GLP attenuated BV2 and primary microglial activation by reducing the pro-inflammatory cytokine expression and promoted the anti-inflammatory cytokine levels.

### GLP modulates LPS- and Aβ-induced microglial migration and morphological changes

**Fig. 2:**
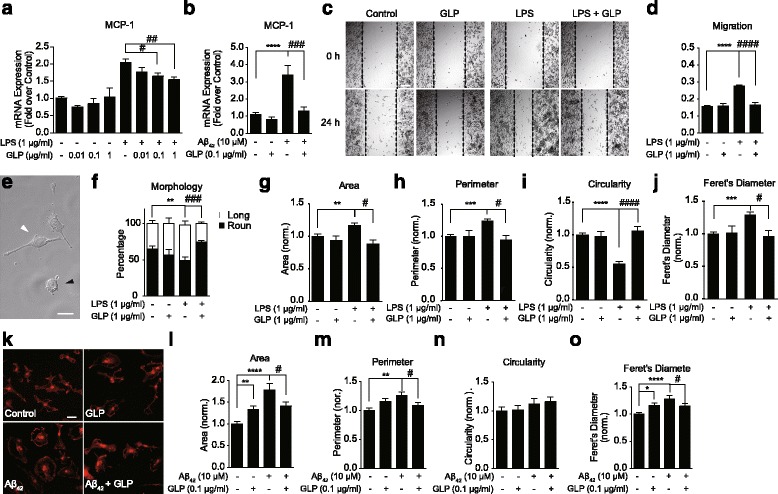
GLP modulates LPS- and Aβ-induced microglial migration and morphological changes. GLP (1 μg/ml) down-regulated the LPS- (**a**) and Aβ-induced (**b**) MCP-1 mRNA expressions in BV2 and primary microglia, respectively. The representative images (**c**) and the quantitative analysis (**d**) illustrated the inhibitory effect of GLP on the LPS-induced BV2 migration. Two major morphological phenotypes were observed within BV2 cell populations (**e**). The ratio of BV2 cell with “round” or “long” morphology was affected by LPS and GLP pre-treatment (**f**). GLP modulated LPS-induced BV2 morphological change in area (**f**), perimeter (**h**), circularity (**i**) and Feret’s diameter (**j**). The representative fluorescent images of primary microglial morphological changes as the results of Aβ stimulation and GLP attenuation (**k**). GLP modulates the Aβ-induced primary microglial morphological changes (**l**–**o**). Values reported were mean ± SEM for at least three independent experiments. One-way ANOVA with Tukey’s multiple comparison test revealed difference from untreated cells (*) and LPS- or Aβ-stimulated condition (#) (compared with control: *p < 0.05, **p < 0.01, ***p < 0.005, and ****p < 0.0001; compared with LPS- or Aβ-stimulated condition: ^#^ *p* < 0.05, ^##^ *p* < 0.01, ^###^ *p* < 0.005, and ^####^ *p* < 0.0001)

The expression of MCP-1 is associated with neuroinflammation and cell motility. We showed increased levels of MCP-1 expressions in BV2 and primary microglia as the results of LPS and Aβ activations, respectively. GLP inhibited such increase of MCP-1 expression (Fig. 2a, b). The reduction in the MCP-1 expression thus might correlate with the attenuated microglial inflammation states and down-regulation of pro-inflammatory cytokine levels. We examined whether the decrease in MCP-1 level leads to changes in microglial morphology and migration (Fig. 2c-j). Our results revealed the LPS-stimulated BV2 migration towards the scratched open area was suppressed by GLP treatment (Fig. 2c, d). Further investigation in BV2 cells observed two major morphological phenotypes, the short-round morphology and the stretched and elongated morphology, here designated as “round” and “long” (Fig. 2e). The untreated BV2 existed mostly round, and under LPS activation, the number of long cells increased significantly. However, pre-treatment with GLP restored the morphologies to round (Fig. 2f). We then carried out more detailed morphological characterisation (Fig. 2g–j). The results revealed that LPS induced increases in cell area, perimeter and Feret’s diameter and a decrease in circularity. These morphological modulations associated with the LPS stimulation were inhibited by GLP pre-treatment. These findings were confirmed in primary microglia (Fig. 2k–o). Activation by Aβ caused microglial morphological changes from ramified to amoeboid with increases in soma sizes. In the presence of GLP, significant inhibitions in the cell area, perimeter and Feret’s diameter measures were detected indicating the modulations of microglial morphologies towards the unstimulated state. It was not clear of the exact reason that GLP treatment alone induced slight increase in the cell area, perimeter and Feret’s diameter. Since no change in the pro-inflammatory cytokine levels were detected, it suggested such changes were unlikely due to inflammatory responses.

### GLP inhibited LPS- and Aβ-induced phagocytosis in microglia

**Fig. 3:**
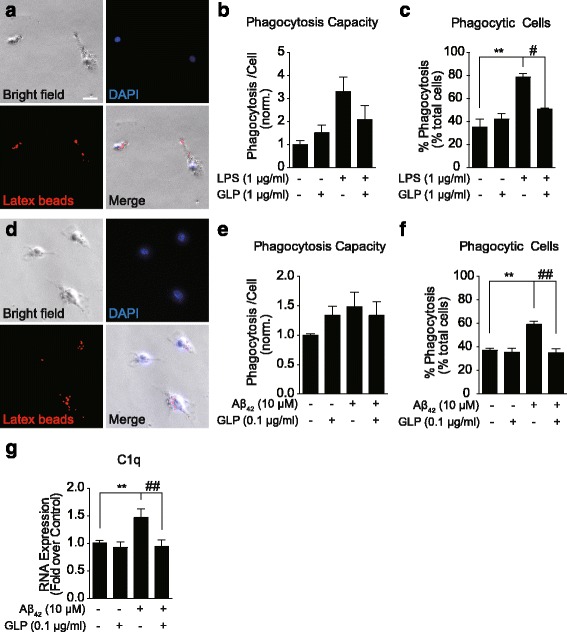
GLP inhibited LPS- and Aβ-induced phagocytosis in microglia. The effect of GLP on the LPS- (**a**–**c**) and Aβ-induced (**d**–**f**) phagocytosis in BV2 (**a**) and primary mouse microglia (**d**), respectively. The phagocytosis capacity was expressed as the ratio of total areas of fluorescent signals to the total numbers of phagocytic cells (**b**, **e**). GLP reduced the proportions of phagocytic cell resulted from LPS and Aβ activation (**c**, **f**). The complement C1q mRNA expression was quantified in primary microglia and GLP inhibited the Aβ-promoted C1q expression (**g**). Values expressed are mean ± SEM for at least three independent experiments. Images of phagocytosis were captured from 16–30 fields per well of cells. Statistical analysis was performed using one-way ANOVA with Tukey’s multiple comparison test, differences from untreated cells (*) and LPS- or Aβ-stimulated condition (#) were revealed (compared with control: **p < 0.01; compared with LPS- or Aβ-stimulated condition: ^#^ *p* < 0.05 and ^##^ *p* < 0.01)

Further, we examined whether, under the same conditions for the inhibited pro-inflammatory cytokines and MCP-1 expressions, GLP modulated microglial phagocytosis. The experiment was conducted using 1-μm fluorescent latex beads in order to induce detectable engulfment by microglia. We demonstrated that in both BV2 and primary microglia, LPS and Aβ activation led to increased number of phagocytic cells, whilst pre-treatment with GLP (1 and 0.1 μg/ml for BV2 and primary microglia, respectively) significantly reduced the phagocytosis events (Fig. 3c, f). We also observed a tendency of changes in the phagocytic capacity in both BV2 and primary microglia (Fig. 3b, e), but the differences were not statistically significant. Cytochalasin D pre-treatment were used as the negative control experiment to completely prevent phagocytosis. Beads attached to the cell surfaces were washed off at the end the phagocytosis assay, and no fluorescent signal was detected from Cytochalasin D pre-treated cells (data not shown). Collectively, our results revealed GLP inhibited the LPS- and Aβ-promoted phagocytosis at 1 and 0.1 μg/ml concentrations, respectively. Under these conditions, the reductions in IL-1β, IL-6 and iNOS expressions were previously observed. In addition, microglial complements are extensively related to phagocytosis and Aβ clearance [31]. In particular, C1q expression was reported to associate to Aβ-induced synaptic loss [31]. We thus examined the expression of complement C1q expression. The results showed an elevated level of C1q expression by Aβ stimulation and reduction by GLP (Fig. 3g). We did not detect significant C1q expression in LPS-stimulated BV2 cells. Previous studies reported that siglec receptors bound to a wide range of sialyloligosaccharides are important regulators of innate immunity [32]. SiglecE and H expressed in mouse microglia were demonstrated to modulate phagocytosis [33–35]. Therefore, we aimed to examine whether GLP modulated phagocytosis in BV2 and primary microglia was through siglec receptors. However, we did not detect expressions of siglecE or H in BV2 cells, which implies that the GLP modulation of phagocytosis might be independent of the siglec receptor mechanism.

### GLP modulates microglial morphology and phagocytosis in vivo

**Fig. 4:**
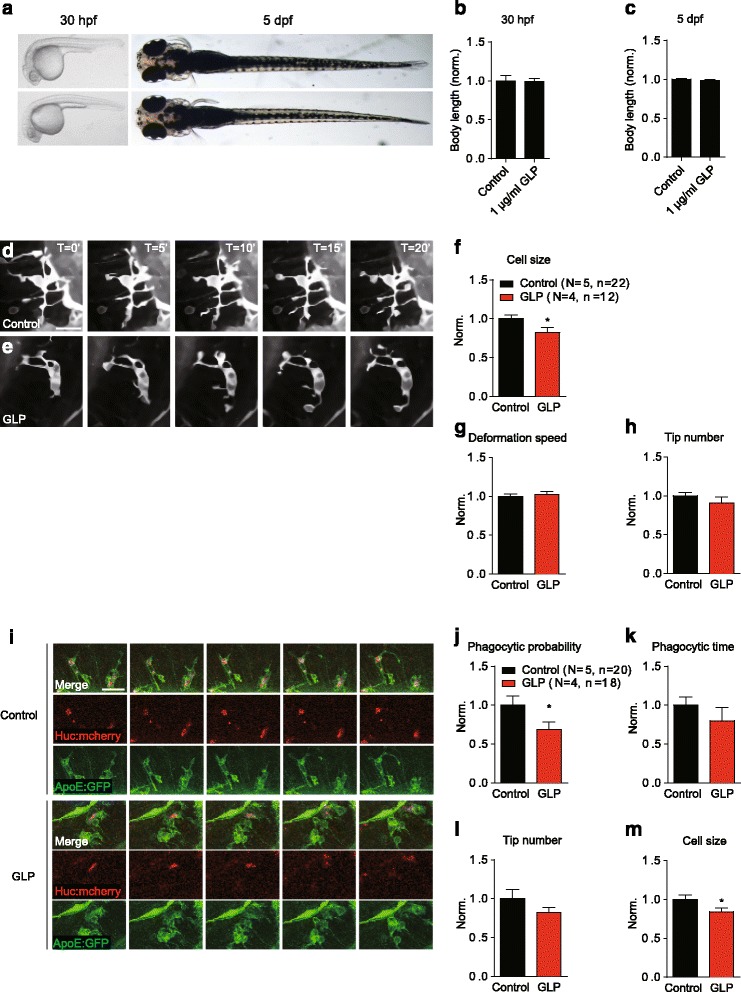
GLP modulates microglial morphology and phagocytosis in vivo. Zebrafish embryo (30 hpf) grown in GLP revealed no developmental defect (**a**). Continued treatment with GLP during the embryonic developmental stage did not affect the larva body lengths (**b**, **c**). Dorsal view of the optic tectum in a 5-dpf *Tg*(*Apo-E:eGFP*, *HuC:mCherry*) zebrafish larvae. Time-lapse images of the dynamics of resting microglia pre-treated with (**e**) or without (**d**) 1 μg/ml GLP. The time scales were in minutes. **f**–**g** The effects of GLP on resting microglial cell sizes, deformation speed and tip numbers. **h** Time-lapse images showing the phagocytic process of microglia *Tg*(*Apo-E:eGFP*, *HuC:mCherry*) zebrafish larvae at 5 dpf. The effect of 1 μg/ml GLP on activated microglial phagocytosis (**j**, **k**) and morphology (**l**, **m**). Data normalised to control. *N* stands for numbers of zebrafish and *n* for the number of cells. Statistical analysis was performed using unpaired two-tailed Student’s *t* test. Values expressed are mean ± SEM. (*p < 0.05)

To confirm the results in vivo, further investigation in the zebrafish brain was conducted in order to determine the role of GLP in microglial behavioural modulations including morphology and phagocytosis changes. A double transgenic lines *Tg*(*Apo-E:eGFP*, *HuC:mCherry*) [29] were used, with Apo-E:eGFP visualising microglia in green and Huc:mcherry labelling neuron in red. The time-lapse images were captured at 5-min intervals for 60 min as previously described [28]. The zebrafish embryonic growth was monitored in the presence of 1 μg/ml GLP from 12 hpf to 5 dpf. No developmental defects were observed (Fig. 4a–c). There are resting and activated microglia in the optic tectum as classified by different reactions in this region [36]. We first analysed the resting microglial morphological dynamics (Fig. 4d–h). Based on previous literature descriptions [37], resting microglia in vivo have “immotile” cell body and many relatively “motile” processes constantly branching out to scan the microenvironment. Our data showed that GLP resulted in a slight decrease in the cell sizes, but the changes in the numbers of branching tips were not significant. The microglial deformation speed, which is defined by difference in cell area between two sequential processes projections, was not affected. For activated microglia in vivo, the cell bodies move fast in the tissue [37]. We analysed the effect of GLP on the activated microglial cell size, tip number and phagocytic properties (Fig. 4i–m). The results showed that GLP decreased activated microglial cell size and phagocytic probability (Fig. 4m, j). However, the effect of GLP on activated microglial tip number and phagocytosis time-length was not significant (Fig. 4l, k).

## Discussion

In the present study, we aimed to investigate the effect of GLP on microglia-mediated neuroinflammation. We first showed that GLP inhibited the LPS-induced pro-inflammatory cytokine expressions in BV2 cell lines. The expressions of IL-1β, IL-6 and iNOS are associated with neurodegenerative diseases. The IL-1β is a potent neuroimmune mediator that takes effect on various cell types including neurons and microglia [38]; iNOS expression is elevated in activated microglia with its synthetic product nitric oxide being one of the major causes of neurodegenerative diseases [39]; and the expression of IL-6 is also strongly linked to AD as an inflammatory mediator [40, 41]. These results thus provide implications that GLP might exhibit neuroinflammation modulatory effects in neurodegenerative diseases such as AD. Therefore, we confirmed this finding in primary microglia in the presence and absence of Aβ activation, and the results were consistent with the responses in BV2 cell. The analysis of IC_50_ values was based on the expression of IL-1β as a representative. The results revealed that GLP seemed to be a more potent inhibitor to Aβ activations in primary microglia compared to BV2 cells when stimulated by LPS. The differences in the IC_50_ values may be attributed to dissimilarities between a cell line and primary cultured microglia, although the different inflammatory stimuli (Aβ and LPS) should also be taken into account. Nevertheless, GLP inhibition of these Aβ-induced inflammatory mediators implies that it might be a potent modulator for AD-related neuroinflammation. In addition, we detected that GLP promoted a significant rise in the anti-inflammatory cytokine TGFβ expression in both BV2 and primary microglia. TGFβ plays crucial role in the inhibition of microglia and macrophage classic activations, the expression of which also enhances the IL-4-induced microglial alternative activation [42]. In combination with previous literature evidence which demonstrated a TGFβ1-dependent clearance of Aβ in microglial cultures [43], our results thus imply that GLP might ameliorate AD pathology. Meanwhile, we also analysed the expression of another anti-inflammatory cytokine arginase-1 (Arg1); we did not detect changes in Arg1 expression. Recently, GLP has been demonstrated to promote NPC proliferation and improve cognitive functions in APP/PS1 transgenic mice, with a significant effect on the Aβ clearance [44]. Therefore, GLP becomes a prominent “dual functional” natural product in targeting AD, possibly via simultaneous actions on both the anti-neuroinflammation and the neurogenesis mechanisms.

As the results of microglial activation, cells display remarkable characteristics in behavioural changes, such as morphology, migration and phagocytosis. Increasing amounts of researches have now focused on to elucidate both the function and morphology of microglia in the healthy and injured brains [45–47]. A recent example in the analysis of microglial morphological and phagocytic activity changes revealed that microglia being the “first responder” after ischemic stroke and subsequent reperfusion process [48]. We observed the LPS-induced BV2 migration towards the open scratched area was significantly blocked by 1 μg/ml GLP. At the same concentration, GLP also showed remarkable inhibitions to the LPS-induced pro-inflammatory cytokine and MCP-1 expressions. In addition, we also recorded during LPS activation BV2 cells elongated and extended from the mostly short and compact morphology to a long shape. This finding coincides with a previous report in which BV2 cells were incubated in the conditioned medium from spinal cord injury for 3–48 h [49]. We provided quantitative descriptions for changes in morphology as decreased cell circularity with increased Feret’s diameter and confirmed that the changes ultimately led to the augmented cell area and perimeter. On the other hand, the quantitative descriptions for primary microglial morphological changes did not entirely follow the observations for BV2. No significant difference was detected in cell circularity under Aβ stimulation, even though there seemed to be an increasing trend. This might be due to the more “irregular” shape of primary microglia. Despite all, increased Feret’s diameter was recorded. As the results of morphology changes, primary microglial cell area and perimeter were remarkably enlarged, which is consistent with BV2 cells. The variations in cell area and perimeter thus seem to be the distinctive features following BV2 and primary microglial activation. Further investigation showed that the LPS- and Aβ-induced morphological changes were almost completely reversed by 1 μg/ml of GLP. In addition, we also studied the effect of GLP on microglial phagocytic behaviour using 1-μm latex beads, since the process of phagocytosis engulfs targets not less than 1 μm. Stimulations with LPS or soluble Aβ oligomer may conceivably mimic the early microglial phagoptosis events. As expected, GLP treatment reduced the LPS- and Aβ-promoted phagocytosis. Our results therefore illustrated GLP modulations on microglial behaviour were associated to MCP-1 expressions. We further extended the investigations of GLP modulations of microglial behaviour in vivo. The zebrafish model used was a double transgenic line, with ApoE:eGFP-labelled microglia in green and HuC:mCherry-labelled neurones in red. Transgenic lines of both labelling were well established [50, 51]. It should be noted that Apo-E was previously described as a zebrafish microglial marker [52, 53]; the *Tg*(*Apo-E:eGFP*) line were then raised in order to reveal zebrafish microglia in green colour [50]. The specificity of this labelling was proved with pU1 morpholino injection, which led to a complete disappearance of Apo-E-positive cells thus confirming the myeloid origin of these labelled cells [50]. In the zebrafish, the cell size, tip numbers and deformation speed are the characteristic features defining resting microglial morphology. GLP reduced the resting microglial cell sizes in vivo, with little impact on the tip numbers and deformation speeds. In BV2, the unstimulated cell morphologies remained unaltered in the presence of GLP. Meanwhile, in the primary microglia, GLP resulted in a slight increase in cell area. Since the pro-inflammatory cytokine levels remained unchanged, it is unlikely that the increase in cell area was resulted from microglial activation. The results of GLP inhibition of activated microglial cell size and phagocytosis in zebrafish were consistent with the findings in LPS- and Aβ-stimulated BV2 and primary microglia.

One other distinguished observation in this study is the complement C1q correlation with oligomeric Aβ stimulation in microglia. This finding is supported by earlier researches [31]. Previously, Stevens et al. [31] demonstrated that microglia is the main source of complement C1q and the expressions of which are specific to the hippocampus and frontal cortex regions in the brain [54]. C1q expression is an essential requirement for the oligomeric Aβ-induced synaptic loss in vivo [55–57]. Deficit in C1q results into an increased number of synapses and provides a neuroprotective function [31, 55]. Our results imply that GLP displayed neuroprotective effect against Aβ oligomers as the treatment with GLP significantly reduced the C1q expression. Furthermore, the neuroprotective effect of GLP might be also achieved through the inhibition of inflammation-induced phagocytosis. Microglial phagocytosis can be considered beneficial under various circumstances. The removal of dead neurones and debris or even excessive live neurones and precursors during adult neurogenesis maintains a homeostatic environment [34, 35]. However, in situations where phagocytosis is stimulated via inflammation, the consequence of which can be detrimental. Although at early-stage phagocytosis may reduce inflammation, excessive removal of pathogens, dead or infected neurones and synapses may lead to phagoptosis of live neurones and synapses [58–60]. Phagocytosis and aberrant phagoptosis are in fact associated with many neurodegenerative diseases such as AD, Parkinson’s disease and frontotemporal degenerations [59, 61, 62]. Mutations in various phagocytosis genes (TREM2, complement receptor 1, CD33, APOE, etc.) were also implicated as risk factors for neurodegenerative diseases [61, 63, 64]. Our in vitro study thus provides implication that GLP may act at early stage of the disease to attenuate inflammation and maintain a controlled phagocytosis event.

## Conclusions

In conclusion, our study provides an insight into the regulatory roles of GLP in LPS- and Aβ-induced microglial behaviour and pro-inflammatory responses. The study stresses the significance of investigation in microglial behavioural response to neuroinflammation. A particular importance is the use of Aβ in order to mimic microglia-mediated neuroinflammation in AD. The results indirectly serve an indication that GLP exhibits a neuroprotective function in the treatment of AD. In conjunction with the neurogenesis effect [44], GLP represents a dual functional cocktail-like natural product, which bares a great potential in the early prevention and treatment of AD.

## Additional file

Additional file 1: Figure S1. GLP exhibited no cytotoxicity and proliferative effect to BV2 cells. (A) GLP effect on BV2 cell viability was examined for 24 h in the presence and absence of LPS stimulation. No cytotoxicity was detected. GLP long-term effect on cell growth was investigated for 48, 72 and 96 h. Cell growth was estimated by total cell count (B) and CellTiter-Glo assay (C). Cells were incubated in normal DMEM culture medium with 10% FBS supplement. Both 1 and 0.01 μg/ml showed no effect on cell growth compared to untreated control. Cells grown in FBS-depleted medium (2% FBS) showed little cell growth, whereas in the FBS-enriched medium (30% FBS), the cell growth exceeds normal cultured medium. **Figure S2.** Confirmation on the integrity of prepared Aβ oligomer. Four microliters of oligomers or fibril were loaded to the nitrocellulose membrane. Dot blot was performed and the oligomer-(A11) and Fibril-specific (ab126468) antibodies were used. In the prepared oligomer (left lane), little fibril fractions were detected, whilst in the prepared Aβ fibril (right lane), certain levels of oligomers were also detected. Staining of 6E10 revealed the total Aβ content. (PDF 207 kb)

## Abbreviations

AD: Alzheimer’s disease
Arg1: Arginase 1
Aβ42: Amyloid beta_42_
CNS: Central nervous system
DMEM: Dubelcco’s Minimal Essential Medium
DMSO: Dimethyl sulfoxide
dpf: Day postfertilisation
E. coli: Escherichia Coli
ELISA: Enzyme-linked immunosorbent assay
FBS: Fetal bovine serum
GLP: *Ganoderma lucidum* polysaccharides
HFIP: Hexafluoroisopropanol
hpf: Hour postfertilisation
HPGPC: High-performance gel permeation chromatography
IL-1β: Interleukin-1 beta
IL-6: Interleukin-6
LB: Luria Bertani
LPS: Lipopolysaccharides
MCP-1: Monocyte chemoattractant protein
NPC: Neural progenitor cell
PBS: Phosphate-buffered saline
qPCR: Quantitative polymerase chain reaction
Tg: Transgenic
TGFβ: Transforming growth factor β
TLR: Toll-like receptor
