# Supplementary material for: Polysaccharides from Ganoderma lucidum attenuate microglia-mediated neuroinflammation and modulate microglial phagocytosis and behavioural response
Source: J Neuroinflammation. 2017 Mar 24;14:63. doi: 10.1186/s12974-017-0839-0 (PMC5364682; doi:10.1186/s12974-017-0839-0)
Supplement: Additional file 1: Figure S1. — GLP exhibited no cytotoxicity and proliferative effect to BV2 cells. (A) GLP effect on BV2 cell viability was examined for 24 h in the presence and absence of LPS stimulation. No cytotoxicity was detected. GLP long-term effect on cell growth was investigated for 48, 72 and 96 h. Cell growth was estimated by total cell count (B) and CellTiter-Glo assay (C). Cells were incubated in normal DMEM culture medium with 10% FBS supplement. Both 1 and 0.01 μg/ml showed no effect on cell growth compared to untreated control. Cells grown in FBS-depleted medium (2% FBS) showed little cell growth, whereas in the FBS-enriched medium (30% FBS), the cell growth exceeds normal cultured medium. Figure S2. Confirmation on the integrity of prepared Aβ oligomer. Four microliters of oligomers or fibril were loaded to the nitrocellulose membrane. Dot blot was performed and the oligomer-(A11) and Fibril-specific (ab126468) antibodies were used. In the prepared oligomer (left lane), little fibril fractions were detected, whilst in the prepared Aβ fibril (right lane), certain levels of oligomers were also detected. Staining of 6E10 revealed the total Aβ content. (PDF 207 kb) [file 12974_2017_839_MOESM1_ESM.pdf]

Supplement Figure 1

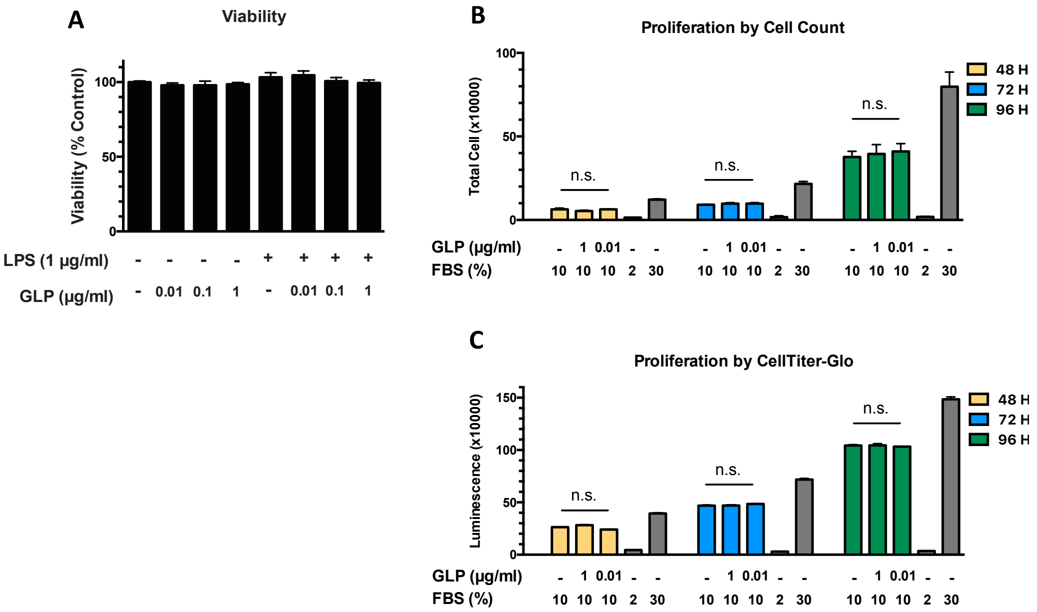

Fig. 1 GLP exhibited no cytotoxicity and proliferative effect to BV2 cells.

(A) GLP effect on BV2 cell viability was examine for 24 hours in the presence and absence of LPS stimulation. No cytotoxicity was detected. GLP long-term effect on cell growth was investigated for 48, 72 and 96 hours. Cell growth was estimated by total cell count (B) and CellTiter-Glo assay (C). Cells were incubated in normal DMEM culture medium with 10%FBS supplement. Both 1 μg/ml and 0.01 μg/ml showed no effect on cell growth compared to untreated control. Cells grown in FBS depleted medium (2% FBS) showed little cell growth, whereas in the FBS enriched medium (30% FBS) the cell growth exceeds normal cultured medium.

Supplement Figure 2

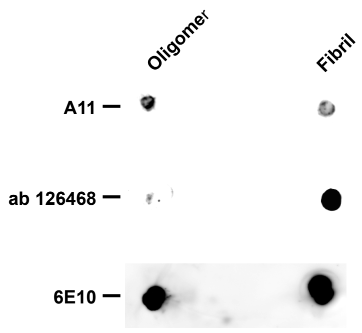

Fig. 2 Confirmation on the integrity of prepared Aβ oligomer

Four microliters of oligomers or fibril were loaded to the nitrocellulous membrane. Dot blot was performed and the oligomer- (A11) and Fibril-specific (ab126468) antibodies were used. In the prepared oligomer (left lane), little fibril fractions were detected, whilst in the prepared Aβ fibril (right lane), certain levels of oligomers were also detected. Staining of 6E10 revealed the total Aβ content.
